# Truncating *CHRNG* mutations associated with interfamilial variability of the severity of the Escobar variant of multiple pterygium syndrome

**DOI:** 10.1186/s12863-016-0382-5

**Published:** 2016-05-31

**Authors:** Ariana Kariminejad, Navid Almadani, Atefeh Khoshaeen, Bjorn Olsson, Ali-Reza Moslemi, Homa Tajsharghi

**Affiliations:** Kariminejad-Najmabadi Pathology & Genetics Centre, Tehran, Iran; Mehrgan Genetics Centre, Sari, Iran; Systems Biology Research Centre, School of Bioscience, University of Skovde, SE-541 28 Skovde, Sweden; Department of Pathology, University of Gothenburg, Sahlgrenska University Hospital, SE-413 45 Gothenburg, Sweden

## Abstract

**Background:**

In humans, muscle-specific nicotinergic acetylcholine receptor (AChR) is a transmembrane protein with five different subunits, coded by *CHRNA1*, *CHRNB*, *CHRND* and *CHRNG*/*CHRNE*. The gamma subunit of AChR encoded by *CHRNG* is expressed during early foetal development, whereas in the adult, the γ subunit is replaced by a ε subunit. Mutations in the *CHRNG* encoding the embryonal acetylcholine receptor may cause the non-lethal Escobar variant (EVMPS) and lethal form (LMPS) of multiple pterygium syndrome. The MPS is a condition characterised by prenatal growth failure with pterygium and akinesia leading to muscle weakness and severe congenital contractures, as well as scoliosis.

**Results:**

Our whole exome sequencing studies have identified one novel and two previously reported homozygous mutations in *CHRNG* in three families affected by non-lethal EVMPS. The mutations consist of deletion of two nucleotides, cause a frameshift predicted to result in premature termination of the foetally expressed gamma subunit of the AChR.

**Conclusions:**

Our data suggest that severity of the phenotype varies significantly both within and between families with MPS and that there is no apparent correlation between mutation position and clinical phenotype. Although individuals with *CHRNG* mutations can survive, there is an increased frequency of abortions and stillbirth in their families. Furthermore, genetic background and environmental modifiers might be of significance for decisiveness of the lethal spectrum, rather than the state of the mutation *per se*. Detailed clinical examination of our patients further indicates the changing phenotype from infancy to childhood.

## Background

Multiple pterygium syndromes (MPS) comprise a group of multiple congenital anomalies of the skin, muscles and skeleton [1, 2]. It is characterised by prenatal growth failure with webbing (pterygium) of the skin present in multiple areas and a lack of muscle movement (akinesia) leading to muscle weakness and severe congenital contractures (arthrogryposis), and scoliosis. The MPS is a clinically and genetically heterogeneous disorder but it is traditionally divided into lethal (LMPS, OMIM 253290), which is fatal before birth or very soon after birth, and non-lethal (Escobar variant, EVMPS, OMIM 26500) MPS types [3]. Escobar syndrome is characterized by short stature, pterygia of the neck, axilla, antecubital, popliteal, digital, and intercrural areas, multiple joint contractures and cleft palate [4]. Dimples at the knees and other joints might be present. Facial features include long face, downslanting palpebral fissures, ptosis, long philtrum, emotionless face, low-set ears, high-arched palate, small mouth, downturned corners of mouth, inability to fully open mouth and retrognathism. Skeletal anomalies such as fusion of cervical vertebrae, scoliosis, kyphosis, flexion contraction of fingers, rocker-bottom feet with vertical talus may be present [5]. Small penis and scrotum and cryptorchidism are seen in males. Females might have aplasia of the labia majora and small clitoris. Variable other features include intrauterine death, congenital respiratory distress, reduced foetal movement, and conductive hearing loss. Changing phenotype from birth to childhood has been observed [6].

Autosomal recessive inheritance appears to be the most common in MPS cases. However, the disease may be transmitted as an autosomal dominant or X linked trait [7–9]. Mutations in genes encoding different subunits of the nicotinergic acetylcholine receptor (AChR), an excitatory cation channel, have been associated with MPS. In humans, muscle-specific AChR is a transmembrane pentameric glycoprotein composed of four different subunits, two α subunits, one β, one δ and one γ/ε subunit, encoded by *CHRNA1*, *CHRNB*, *CHRND* and *CHRNG*/*CHRNE*, respectively. The AChR exists in two forms, the embryonic form, present in foetal and denervated muscle and the adult form, which is predominantly expressed postnatal [10, 11]. In humans, the switch from the embryonic AChR to the adult is apparently completed by the 33st week of gestation, in the late foetal and perinatal period [12]. The γ subunit of AChR (*CHRNG*) is expressed during early foetal development, whereas in the adult it is replaced by a ε subunit (*CHRNE*). Each AChR subunit comprises a large extracellular glycosylated N-terminal ligand-binding domain, followed by three hydrophobic transmembrane regions, which form the ionic channel, followed by an intracellular region of variable length. A fourth hydrophobic transmembrane region is located at the C-terminal domain.

Hereditary congenital myasthenic syndrome (OMIM 608931, 608930, 601462 and 254210) is predominantly caused by mutations in *CHRNA1, CHRNB1, CHRND* and *CHRNE*, the genes encoding α, β, δ and ε AChR subunits, respectively [13]. While mutations in the gene encoding the gamma subunit of the AChR (*CHRNG*), cause most cases of EVMPS and a smaller percentage of cases of LMPS, homozygous nonsense mutations in *CHRNA1* and *CHRND*, are associated with LMPS [4, 14–17].

Whole-Exome Sequencing (WES) was applied in 9 Iranian cases (2 foetuses and 7 children) with clinical presentation of arthrogryposis. In 4 cases *CHRNG* was suspected as the most likely gene underlying the disease and the association of *CHRNG* with the disease was confirmed in three out of four cases. Here, we report one novel and two previously reported deletions of two nucleotides in *CHRNG*, causing a frameshift predicted to result in premature termination of the foetally expressed gamma subunit of the AChR in three families of Iranian descent with EVMPS. We also emphasize the changing phenotype from infancy to childhood and intrafamilial variability.

### Cases

**Fig. 1:**
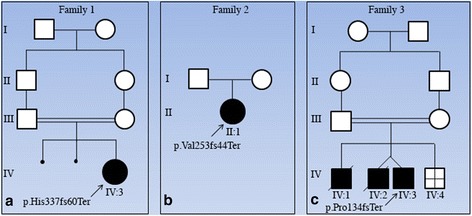
Pedigrees for families included in this study. Affected individuals are represented with shaded symbols and probands are indicated with an arrow. Identified mutation is indicated in each family

**Fig. 2:**
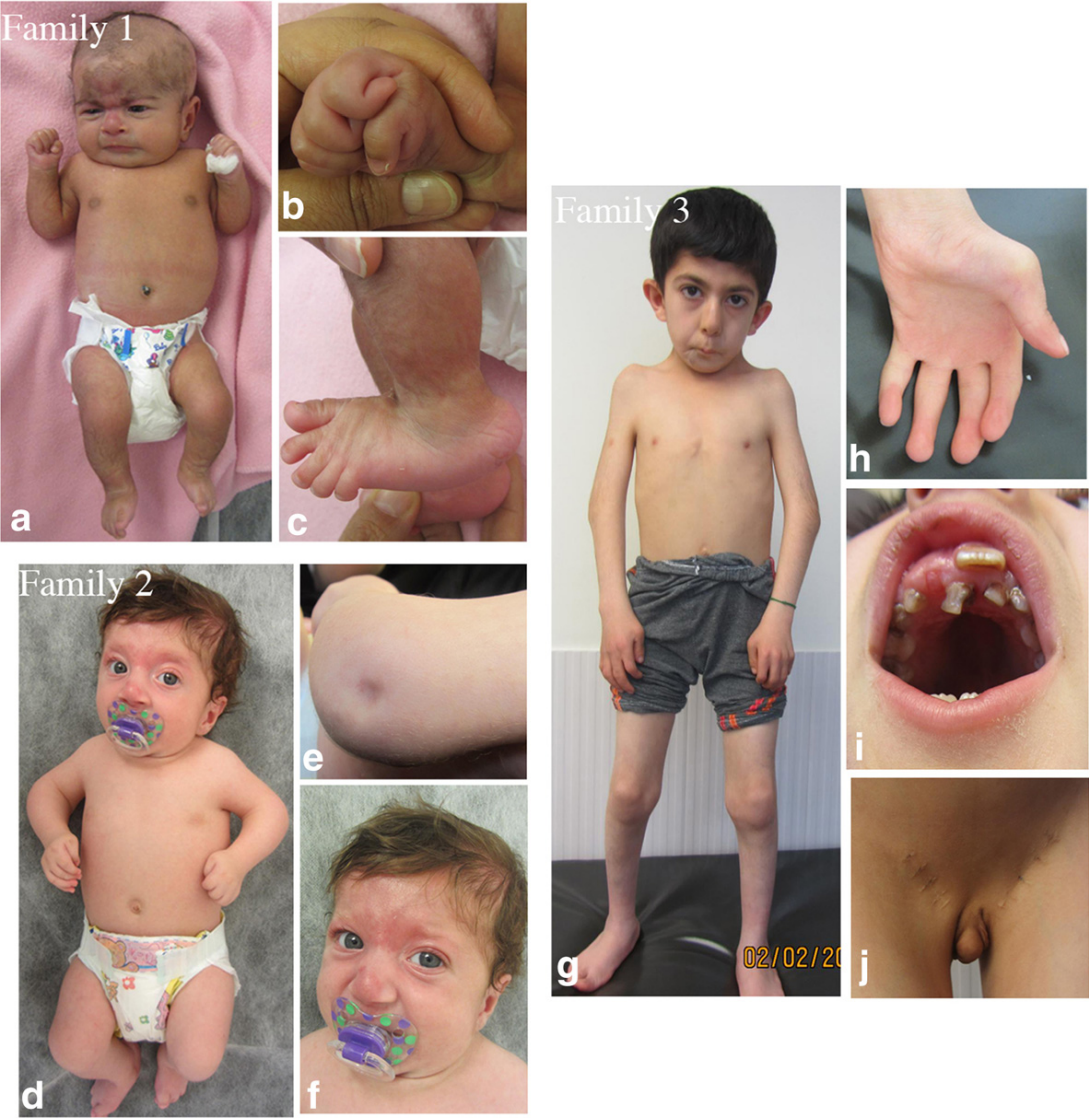
Clinical features of subjects with CHRNG mutations and non-lethal variant of MPS. Case IV:1 from family 1, demonstrates a very short neck, mild pterygia in the axillae, elbows and knees, contracture of joints, clenched hands with thumbs held across palm and club feet, at 1 month of age. The elbows and knees were held in flexed position and had limitation of movement (**a** and **b**). She had rockerbottom feet (**c**). In family 2, individual II:1 has multiple joint contractures in the neck, shoulders, elbows, wrist, fingers, knees and halluces were noted (**d**) and dimpling in the elbows (**e**). She has a capillary hemangioma on nasal nip and forehead, and micrognathia in the face (**f**). In family 3, individual IV:3 at the age of 7 years demonstrates facial dysmorphism including posterior and anterior low hairline, down-slanting palpebral fissures, epicanthal folds, broad nose, high nasal bridge, long philtrum, high-arched palate, rockerbottom feet, and micrognathia (**g**). He has camtodactyly of all fingers and skin syndactyly of fingers (**h**) and the teeth are small and malpositioned (**i**). The micropenis is apparent (**j**)

In family 1 (Fig. 1a), a female (IV:3) was born to healthy consanguineous (first-cousin) parents of Iranian descent. The mother had history of two spontaneous abortions at 6 weeks and 7 weeks of gestation. Pregnancy was uneventful. Amniocentesis was performed because of high risk of trisomy 21 in the first trimester screening. Chromosomal study was normal. Elective cesarean section was performed at term. At birth, her weight was 2800 g (10^th^ centile), and her length and head circumference were 44 cm (<3^rd^ centile) and 35 cm (50^th^ centile), respectively. Physical examination at 1 month of age disclosed a very short neck, mild pterygia in the axillae, elbows and knees, contracture of joints (elbows, wrists, fingers, knees and ankles) clenched hands with thumbs held across palm and club feet (varus). The elbows and knees were held in flexed position and had limitation of movement (Fig. 2a, b). She had rockerbottom feet, with almost no movement in ankles (Fig. 2c). Facial dysmorphism included hemangioma over forehead and nose, strabismus, flat nasal bridge, downturned corners of mouth. Whole body X-rays did not show major abnormality.

In family 2 (Fig. 1b), individual II:1 was the first and only child of a unrelated couple. The mother noted reduced foetal movement during pregnancy. Delivery was at 39 weeks of gestation by cesarean section because of breech position. Birth weight, length and head circumference were 3150 g (<50^th^ centile), 43 cm (<3^rd^ centile) and 36 cm (75^th^ centile), respectively. When assessed at two and a half months, her weight was 4500 g (<10^th^ centile), height 53 cm (<3^rd^ centile) and head circumference 41.5 cm (<75^th^ centile). Multiple joint contractures in the neck (torticolli), shoulders, elbows, wrist, fingers, knees and halluces were noted (Fig. 2d). The hands were clenched and thumb held across palm. The shoulders were rounded, sloping and decreased in muscle mass. There was dimpling in the elbows (Fig. 2e). Pterygium was noted in the axillary region. She had a capillary hemangioma on nasal nip and forehead, and micrognathia in the face (Fig. 2f). Full body X-ray showed scoliosis. Echocardiography was normal. Ultra-sound examination of the brain, abdomen (liver, gall bladder, pancreas and spleen), kidneys and hip joint appeared normal.

In family 3 (Fig. 1c), individual IV:3 was born to first-cousin parents of Iranian descent. The first pregnancy was a boy (IV:1) with multiple contractures and pterygium in joints, who died at the age of 6 months. The second pregnancy was a twin pregnancy, the proband (IV:3) and a similarly affected boy (IV:2) who died at the age of 1.5 years. The third pregnancy resulted in intra-uterine-foetal death at 20 weeks of gestation (IV:4). Delivery was by caesarean section at 9 months of pregnancy. Birth weight was 2500 g (5^th^ centile). Pterygium in knees and elbows was noticed from birth. The infant had multiple episode of epistaxis, which led to the diagnosis of von Willebrand type III hemophilia. Milestones were delayed; he held his head at age 1 year, he stood at 2 years of age, and walked at age 2.5 years. Cognition was normal and he started saying words at age 10 months and he had normal speech at 7 years of age. Bilateral inguinal hernia was treated surgically at the age of 3 years old. At 7 years of age, his height, weight and head circumference were 117 cm (<25^th^ centile), 16 kg (<3^rd^ centile) and 49.5 cm (<50^th^ centile), respectively. Detailed review at the age of 7 years revealed facial dysmorphism including posterior and anterior low hairline, down-slanting palpebral fissures, epicanthal folds, broad nose, high nasal bridge, long philtrum, high-arched palate, and micrognathia (Fig. 2g). The teeth were small and malpositioned (Fig. 2i). Pterygia were present in neck, axillae, antecubital and in intercrural region. Contracture at elbows, limitation of elbow extension, limited movement of flexion and extension in wrists, camtodactyly of all fingers and skin syndactyly of fingers, rockerbottom feet, and second toe overlapping first and third in both feet, limited movement in ankles, limited flexion and extension of knees and micropenis were apparent (Fig. 2g, h, j). Intellectual development appeared normal. Clinical features in the investigated families are summarised in Table 1.

**Table 1:** Features in investigated families affected by non-lethal EVMPS

	Case IV:3 (Family 1)	Case II:1 (Family 2)	Case IV:3 (Family 3)
Consanguinity	+	-	+
Feature			
History of abortion/IUFD	+	-	+
Reduced fetal movement	-	+	-
Sex	F	F	M
Age at examination	1 month	2.5 months	7 years
Short stature	+	+	-
Multiple contractures	+	+	+
Pterygium axilla	+	+	+
Pterygium elbows	+	-	+
Pterygium knees	+	-	+
Camptodactyly	+	+	+
Clenched hands	+	+	-
Thumb crossing palms	+	+	-
Clubfeet	+	+	-
Rockerbottom feet	+	-	+
Expressionless face	-	-	+
micrognathia	+	+	+
Downturned corners of mouth	+		
Low set ears	-	+	-
Downturned corners of mouth	+	-	-
High arched palate	-	-	+
Webbed neck/short neck	+	+	+
Long philtrum	+		+
Hearing loss	NE	NE	-
Cryptorchidism	-	-	+
Other	Strabismus	Dimpling of elbow, hemangioma on forehead and nose,	Inguinal hernia Von willebrand hemophilia

## Methods

### DNA isolation

Blood samples were collected from probands, parents and siblings. Extraction of genomic DNA was performed from whole blood from patients and parents, using DNeasy Blood & Tissue kit (Qiagen, Hilden Germany), according to the manufacturer’s instructions. In addition, genomic DNA was extracted from 120 Iranian blood donors, who served as controls.

### Genetic analysis

#### Exome sequence analysis

The WES was performed on DNA from patients and their unaffected parents, as previously described [18]. Briefly, target enrichment was performed with 3 μg genomic DNA using the Sure SelectXT Human All Exon kit version 5 (Agilent Technologies, Santa Clara, CA, USA) to generate barcoded whole-exome sequencing libraries. Libraries were sequenced on the HiSeq2000 platform (Illumina, San Diego, CA, USA) as paired-end 2 × 100-bp reads with 60x coverage. Quality assessment of the sequence reads was performed by generating QC statistics with FastQC (http://www.bioinformatics.bbsrc.ac.uk/projects/fastqc). Read alignment to the reference human genome (hg19, UCSC assembly, February 2009) was done using BWA [19] with default parameters. After removal of polymerase chain reaction (PCR) duplicates (Picard tools, http://picard.sourceforge.net) and file conversion (samtools [20]) quality score recalibration, indel realignment and variant calling were performed with the HaplotypeCaller algorithm in the GATK package [21] based on established best practices [22].

#### Variant annotation and selection

Variants were annotated with ANNOVAR [23] using a wide range of databases such as dbSNP build 135, dbNSFP, KEGG, the Gene Ontology project and tracks from the UCSC. A filtering strategy, directed to disease gene candidates, was performed by QIAGEN’s Ingenuity® Variant Analysis™ software (www.qiagen.com/ingenuity) from QIAGEN Redwood City. We focused on exonic variants where the mutation produced a missense change, stop gain or stop loss. We required at least two mutations in the same gene for further analysis. Only those changes that were predicted to be damaging or with unknown impact were kept. We excluded SNPs that were shared with our control dataset (>1 % in dbSNP [24], the Exome Variant Server (NHLBI)(http://evs.gs.washington.edu/EVS/), the 1000 Genome Project Database and the human Background Variant Database (http://neotek.scilifelab.se/hbvdb/)) as well as those labeled as compound heterozygous.

#### Polymerase chain reaction (PCR) and Sanger sequencing

The variants found by WES in the candidate genes were examined in the individuals by PCR and Sanger sequencing using an ABI 3730XL (GATC Biotech, Constance, Germany and Eurofins MWG Operon, Ebersberg, Germany) if they had either a variant frequency <1 % in the healthy population or a minor allele frequency (MAF) below the normal MAF in the European population in EVS. PCR was performed on DNA samples from patients and their unaffected parents. PCR primers are available on request.

## Results

### Genetic findings

**Fig. 3:**
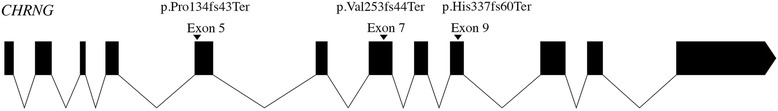
*CHRNG* mutations in the investigated families with EVMPS

Data from WES on DNA from patients and their parents were analysed through the use of the Ingenuity Variant Analysis (IVA) software (Qiagen, Hilden Germany). The filtering strategy narrowed the starting variants to 16, 11 and 12 genes in case IV:3 (family 1), II:1 (family 2) and IV:3 (family 3), respectively. This approach allowed the identification of homozygous deletion of two nucleotides in exons of *CHRNG* in these cases. All other related disease-causing genes were excluded in these cases. We identified a novel homozygous deletion of two nucleotides in exon 9 (c.1009-1010delCA) (ID SUB1128405) of *CHRNG* in case IV:3 of family 1, leading to a frameshift predicted to result in premature termination (p.His337fs60Ter) (Fig. 3). In case II:1 of family 2, the filtering strategy narrowed the variants to 11 genes, including a homozygous previously reported deletion of two nucleotides in exon 7 (c.753-754delCT) of *CHRNG*, leading to a frameshift predicted to result in a premature termination (p.Val253fs44Ter) (Fig. 3). In case IV:3 of family 3, a previously reported homozygous deletion of two nucleotides in exon 5 (c.401-402delCT) of *CHRNG* was identified, leading to a frameshift. The c.401-402delCT mutation was predicted to result in premature termination (p.Pro134fs43Ter) (Fig. 3).

Putative deleterious variants in *CHRNG* exons 5, 7 and 9, were confirmed in the children and their parents by PCR and Sanger sequencing analysis. The unaffected parents were heterozygous for the two nucleotide deletions. None of the mutations was reported in public databases as a polymorphism. However, in order to define the frequency of the identified variants in population-matched controls, PCR and Sanger sequencing of *CHRNG* exons 5, 7 and 9 was performed in 120 Iranian blood donors, who served as controls. The results from sequencing analysis excluded the *CHRNG* sequence variants in 120 Iranian control individuals.

The recurrent homozygous deletion of two nucleotides in exon 7 (c.753-754delCT) was identified in the only child of a unrelated couple (in case II:1 of family 2). Haplotyping was not available so it is not known if this was due to a founder mutation. However, the percentage of homozygosity in exome sequencing of this case compared to two other probands born to the first-cousin parents was notably lower (5.6 %).

## Discussion

The mammalian muscle-specific acetylcholine receptor is a transmembrane glycoprotein with two alpha, one beta, one delta and one gamma (expressed in foetal and denervated muscle) or epsilon (expressed in adult skeletal muscle) subunit [12]. The gamma subunit of AChR, encoded by *CHRNG*, is expressed prior to the thirty-third week of gestation in humans [10, 25, 26]. It helps to establish the primary encounter of muscle and axon and has thus, a pivotal role in neuromuscular organogenesis and ligand binding [27]. Mutations in gamma subunits cause MPS, either the severe and fatal form (LMPS), or the milder and non-lethal Escobar variant [4, 14, 15]. Previous study has demonstrated that disruption of gamma subunit expression prevents the correct localization of the receptor in cell membranes [14]. The ultimate result of the impaired γ subunit structure is reduced prenatal muscle strength and movement, explaining the contractures and pterygia [14].

Here, we report three families with EVMPS associated with one novel and two previously reported frameshift mutations in *CHRNG*. These mutations are anticipated to result in premature mRNA nonsense-mediated decay followed by a γ subunit deficiency or predicted to result in premature termination of transcription of the foetally expressed gamma subunit of the AChR. All patients had clinical features consistent with MPS Escobar variant, including arthrogryposis multiplex congenita, multiple pterygia, short stature and dysmorphic facial features, corresponding to the developmental deformities in utero.

The novel homozygous deletion (c.1009-1010delCA; p.His337fs60Ter) in family 1 is located in exon 9 of *CHRNG.* In the absence of nonsense-mediated decay, the mutation is predicted to result a frameshift followed by a truncated gamma AChR subunit lacking intracellular region and the fourth hydrophobic transmembrane domain. Consequently, the mutation will lead to a dysfunctional or deficient foetal AChR, which in turn will cause impaired prenatal neuromuscular transmission and organogenesis followed by developmental defects. It is likely that this homozygous mutation caused the history of abortions in this family. Since the abortions occurred very early in pregnancy (at 7 and 8 weeks of gestation, respectively), the fetopathological examination could not be performed and therefore, there are no further clinical details. It is thus uncertain whether the typical clinical features corresponding to the lethal MPS were present in these cases.

The recurrent c.753-754delCT, pVal253Alafs44Ter mutation identified in family 2 was previously reported in patients from different ethnic backgrounds affected with both the lethal and non-lethal phenotypes [15, 17]. It was found in the homozygous state and in combination with a second *CHRNG* heterozygous mutation. The previously reported homozygous c.753-754delCT, pVal253Alafs44Ter mutation was found in cases with the lethal and non-lethal MPS, born to consanguineous parents of Turkish descent [15, 17]. It was also found together with a second *CHRNG* heterozygous mutation in two compound heterozygous individuals with EVMPS phenotype [15]. Thus, the c.753-754delCT mutation may cause both lethal and non-lethal MPS, and the homozygous state of the mutation is not decisive of the lethal spectrum. Furthermore, there is no apparent correlation between mutation position and clinical phenotype. Therefore, genetic background and interindividual differences, and environmental modifiers might be of significance.

As previously reported, severity of the phenotype varies significantly both within and between families with MPS [6, 15]. This is further supported in family 3, in which the first and second siblings with non-lethal MPS were severely affected and died in the early infancy. However, it is unclear how the lethality in the two affected siblings was influenced by the severity of the congenital deformities. As previously suggested [15] the chance of a non-lethal MPS phenotype arising in further affected siblings within families associated with homozygous c.401-402delCT; p.Pro134Argfs34Ter mutation is more likely. The c.401-402delCT; p.Pro134Argfs34Ter mutation was previously reported in a 14-year-old boy with clinical features closely resembling individual IV:3 of family 3 [4]. Contracture and pterygia in all major joints and facial features characteristic for Escobar syndrome was present in both cases. However, bilateral inguinal hernia and micropenis observed in our patient, was not reported in the previous case. This homozygous deletion mutation was further reported in an individual with EVMPS phenotype born to a non-consanguineous couple from the UK [15]. The frequently observed *CHRNG* c.753-754delCT; pVal253Alafs44Ter and c.401-402delCT; p.Pro134Argfs34Ter mutations indicate the mutational hotspot of these residues [4, 15, 17].

It has been suggested that there is a high concordance between siblings regarding the severity of clinical findings in families with EVMPS [15]. However, the history of increased frequency of abortions and stillbirth in reported families with EVMPS [4, 14, 15] and the presence of two abortions in family 1 and two perinatal deaths in family 3 suggest that variable severity of disease could exist in the same family.

Given the fact that *CHRNG* is expressed only during early foetal development and in denervated cells and that the γ subunit is not a component of the adult acetylcholine receptor [10–12], postnatal muscle biopsy from affected individuals with EVMPS phenotype is inapplicable to assess the effect of *CHRNG* mutations on transcripts and protein levels. Likewise, it is unlikely that postnatal muscle biopsy discloses specific muscle histopathology, as postnatal muscle weakness is not a feature of *CHRNG* associated MPS Escobar variant. Although myasthenic features and abnormal muscle histopathology is not expected in patients with *CHRNG* mutations, congenital diaphragmatic muscle weakness, diffuse myopathy and myasthenic-like features have frequently been reported in some patients [14]. This can be due to the role of γ subunit AChR in muscle organogenesis.

Clinical examination of our patients indicates that the complete clinical features of MPS Escobar variant develop past infancy. This is supported by previous study describing detail clinical features of individuals with EVMPS [6]. While certain clinical features are not present at birth and become apparent, as the infant grows older, other features improve with time. For example, the clear pterygia is not apparent in cases IV:3 (family 1) and II:1 (family 2), at the age of one and two and a half months, respectively, whereas it is completely evident in case IV:3 (family 3), at the age of seven years. Infants have the severe joint contractures and limited movement at birth, which persists but might improve as the child grows older. Many infants born with Escobar syndrome have clenched hands with the thumb held across the palm. However, they can open their fist and have improvement of the position of the thumb as they grow older [6]. Individuals IV:3 and II:1 in family 1 and 2, respectively, show clenched hands with the thumbs held across thumb. Likewise, individual IV:3 in family 3 is said to have had the similar clinical finding at infancy. However, this clinical condition has improved and he only has camptodactyly at the age of 7 years. Moreover, the characteristic facial appearance of Escobar syndrome including down-slanting palpebral fissures, ptosis, long philtrum, sad and emotionless face, observed in the older child is not present in the infants with Escobar syndrome. Facial features dominantly present at birth include epicanthal fold, low-set ears, and micrognathia. Capillary hemangioma observed in individuals IV:3 and II:1 in family 1 and 2, respectively, is additional clinical feature that has been reported in infants with Escobar syndrome, but it is absent in the older child with Escobar syndrome. Taking together, accurate diagnosis and prognosis require follow-up of infants presenting multiple joint contractures or arthrogryposis multiplex congenital at birth, as many of them develop the characteristic features of Escobar syndrome MPS over the first few years of life.

## Conclusions

In conclusion, we present three families affected by non-lethal MPS and a novel and two previously reported homozygous frameshift truncated mutations in *CHRNG* predicted to result in truncations. We suggest that there is no apparent correlation between mutation position and clinical phenotype. Our data suggest the changing phenotype from infancy to childhood in individuals affected by MPS Escobar variant and that severity of the phenotype varies both within and between families with MPS.

### Web resources

Following Databases were used in this study:

- The Exome Variant Server: NHLBI Exome Sequencing Project (ESP), Seattle, WA;
- URL: http://evs.gs.washington.edu/EVS/
- 1000 Genome Project Database: http://browser.1000genomes.org/index.html
- Human Background Variant DataBase: http://neotek.scilifelab.se/hbvdb/

## Abbreviations

AChR, acetylcholine receptor; EVMPS, escobar variant of multiple pterygium syndrome; LMPS, lethal form of multiple pterygium syndrome; MPS, multiple pterygium syndromes; MAF, minor allele frequency; PCR, polymerase chain reaction; WES, whole-exome sequencing; EVS: exome Variant Server
